# Scoping Review: Medical Education Interventions Optimizing Social Workers in the Emergency Department

**DOI:** 10.5811/westjem.2022.10.55693

**Published:** 2022-12-30

**Authors:** Tehreem Rehman, Harrison Pidgeon, Brock Chimileski, Shelby Parker, Dennis Hsieh

**Affiliations:** * University of Colorado School of Medicine, Department of Emergency Medicine, Aurora Colorado; † Icahn School of Medicine at Mount Sinai, Department of Emergency Medicine, New York, New York; ‡ Yale School of Medicine, Department of Emergency Medicine, New Haven, Connecticut; § University of Rochester-Strong Memorial Hospital, Department of Emergency Medicine, Rochester, New York; || Contra Costa Health Plan, Martinez, California

## Abstract

**Introduction:**

As the significance of social workers (SW) in improving healthcare delivery in the emergency department (ED) continues to expand, emergency physicians will increasingly be expected to effectively partner with SWs in both academic and community settings. In this scoping review we sought to provide evidence-based recommendations for effective emergency clinician educational interventions on how to incorporate SWs in the ED to address health-related social needs while also identifying directions for future research.

**Methods:**

We conducted a systematic literature review of publications in PubMed, CINAHL, Cochrane Database of Systematic Reviews, Cochrane Central Register of Controlled Trials, and APA PsycINFO. A search strategy was designed in accordance with Peer Review of Electronic Search Strategies (PRESS) guidelines. Using the scoping review framework by Arksey and O’Malley, we applied consensus-based inclusion and exclusion criteria to guide study selection. A Preferred Reporting Items for Systemic Reviews and Meta-Analyses (PRISMA) flow chart delineating the selection process was generated using Covidence.

**Results:**

Our search strategy identified nine qualifying articles for further analysis out of an initial sample of 2,119 articles. Of the nine articles that underwent full text review, 89% (8/9) evaluated a short educational didactic with or without a hands-on component to reinforce learning. Barriers to successful implementation of curricula discussed in all articles included time constraints, lack of buy-in from clinical faculty, lack of knowledge of appropriate referral sources once a problem is identified, and perceived distraction of the training from more standard clinical topics. Facilitators of curricula implementation and training success included the presence of a pre-existing and structured weekly conference schedule, ability to complete the training in a relatively short time frame or during intern orientation, presence of simulation resources, and residents’ overall perceived interest in the topics.

**Conclusion:**

Ultimately, we found that interdisciplinary learning with SWs is generally well received by participants, and we offer various suggestions on incorporation into student and resident education. Moving forward, we recommend that a standardized curriculum of working with SWs be developed using didactic sessions, simulation, and/or direct observation with feedback.

## INTRODUCTION

For more than 60 years, the value of social workers (SW) in medicine has been recognized.^1^ The emergency department (ED) requires a multidisciplinary, team-based approach in which SWs are a vital component.^2^ Although many academic EDs employ SWs and care managers, there is a lack of standardized training for medical students, residents, attending physicians and other clinicians in the ED on how to effectively incorporate SWs into the patient care team.

As the significance of SWs in improving healthcare delivery in the ED continues to expand, particularly with respect to lowering costs, increasing patient satisfaction, improving quality, and reducing physician burnout, emergency physicians will increasingly be expected to effectively partner with SWs in both academic and community settings.^3^ The SW scope of practice encompasses a wide range of services, including discharge assistance and counseling. A holistic approach renders SWs particularly valuable in addressing health-related social needs in the dynamic. safety-net setting of an ED.^3^ In this literature review and scoping framework we sought to provide evidence-based recommendations for effective ED clinician educational interventions on how to incorporate social workers in the ED to address patients’ health-related social needs while also identifying directions for future research.

## METHODS

While serving on the Emergency Medicine Residents’ Association (EMRA) Social Emergency Medicine (EM) Committee, one of the authors of this study (TR) created a working group to improve education in social EM. Specifically, the purpose was to investigate existing literature related to educating residents and medical students on ED care models that include SWs and care managers, and to create resources to assist members in implementing multidisciplinary care models as part of their training programs. Using Peer Review of Electronic Search Stratregies (PRESS) guidelines, we conducted a systematic literature review in PubMed, CINAHL, Cochrane Database of Systematic Reviews, Cochrane Central Register of Controlled Trials, and APA PsycINFO.^5^ We developed the search threads after categorizing the four necessary elements of our research question: curriculum; students; social work; and emergency setting. The table illustrates the search terms used.

We established inclusion and exclusion criteria. Two authors (TR and HP) reviewed respective abstracts for potential relevance based on search results. The same two authors achieved consensus after resolving differences through real-time rigorous comparison of articles to predefined inclusion and exclusion criteria. Two other authors (TR and HP) reviewed the full text of selected abstracts and independently assessed their relevance. For any disagreements, all four authors convened for real-time comparison to predefined inclusion and exclusion criteria. This process led to a group consensus for a final decision for all remaining full-text articles. The reference section for each included article was checked for additional articles that were otherwise missed in the initial search.

We used the web-based tool Covidence (Melbourne, Australia) to facilitate study selection. We performed the final two steps of sorting and summarizing collected data after collectively establishing the categorization scheme. We organized articles by training level, educational strategy, evaluation methods, outcomes, facilitators to implementation, and barriers to implementation. The purpose of this categorization scheme was to provide guidance on best practices for replication of the studied educational interventions. The results of our literature search are presented in a PRISMA flow chart in the figure.

## RESULTS

Of an initial sample of 2,119 articles, our search strategy identified nine qualifying articles for further analysis. No additional articles were detected after searching the references of the selected nine articles. The educational strategies, outcomes, and barriers to implementation discussed in these articles are summarized in a table including links to each paper that are included in the Appendix.[Table t1-wjem-24-201]

Although a limited number of articles were included in the final review, we found a wide range of curricula structure, levels of time investment, and deliverables to sustain long-term impact of the educational interventions. Four of the nine articles shared a similar curricular design of an introductory didactic session followed by varying mechanisms of hands-on practice with the new skill.^6^–^9^ Four additional articles described the use of a didactic model alone of at least one training session without hands-on practice.^10^–^13^ The remaining article described use of hands-on training alone.^14^

Most articles described simulation cases or interactive case review. Four articles described involvement of direct patient interaction.^7^,^9^,^12^,^14^ Three of these were directly integrated into regularly scheduled clinical shifts.^9^,^12^,^14^ Five articles reported training time allotments between 20 minutes to three hours.^6^,^8^,^10^,^11^,^13^ Other articles did not clearly report time requirements. Another identified educational strategy was the development of pocket-sized reference cards for participants to use for long-term reinforcement of the training.^6^,^10^

The included studies all entailed interdisciplinary training. Most of the included studies directly involved EM residents and/or attending physicians. Only one article reported training of medical students.^8^ All studies included SWs or SW students as direct contributors to curricula development, execution, and/or attendance. Seven studies involved at least one additional specialty, such as nursing, pharmacy, or other ED staff.

Studies included evaluations of the impact of the medical interventions on trainees. Seven studies used pre- and post-intervention surveys as their primary means of analysis, most commonly assessing for self-reported confidence in the skill in question. One study objectively assessed competence in the new skill.^9^ Social workers directly evaluated participants in two articles.^9^,^14^ Results of each article were positive, with residents frequently reporting improved confidence or knowledge on the topic.

## DISCUSSION

Working on the front lines, emergency physicians become intimately familiar with health-related social needs when providing optimal care to patients. With growing recognition of the importance of interdisciplinary training, the successful incorporation of SWs into medical education has been reported in several instances in the literature. Through this scoping review, we were able to derive a framework of barriers and facilitators to guide implementation of similar educational interventions at other institutions. Of the articles that underwent full text review, 89% (8/9) described a short educational didactic with or without a hands-on component to reinforce learning. Short educational modules were likely implemented within the current paradigm of Accreditation Council for Graduate Medical Education-protected academic time, which most EM programs group as a five-hour continuous didactic time.

Barriers to successful implementation of such curricula included time constraints for new material within already established resident conference schedules, lack of buy-in from clinical faculty, lack of knowledge of appropriate referral sources once a problem is identified, and perceived distraction of the training from more standard clinical topics. Facilitators of curricula implementation and training success included the presence of a pre-existing and structured weekly conference schedule (thus reported as both a barrier to and a facilitator of implementation), ability to complete the training in a relatively short time frame or during intern orientation, presence of simulation resources, and residents’ overall perceived interest in the topics.

Opportunities for inclusion of social work professionals in the medical education environment abound. Resident physicians are required to participate in weekly didactic activities including lectures, labs, asynchronous learning, simulations, grand rounds, or other forms of education that are often consolidated into a weekly conference day in which residents are not responsible for clinical duties during this protected learning time. As seen in the studies reviewed here, SW involvement in didactics was well received by resident learners, particularly in simulation scenarios and case-based learning.^6^–^7^,^10^ Social workers could be recruited by organizers of residency education to host lectures or workshops on topics that they commonly deal with in the ED (eg, patient housing instability, trauma-informed care, substance use disorder/addiction) as well as lead simulation cases for residents to practice working with SWs. Similarly, medical students in the clinical stage of training could participate in this type of case-based learning either during dedicated didactic sessions or while rotating in the ED alongside the residents.

Future investigation on the most effective approach to implementation and extent of education during training is warranted, as no identified studies compared different educational models. Additionally, we found significantly more data in the literature pertaining to residents and attending physicians than to medical students. This highlights the need for greater studies on SW involvement in the training of medical students. Medical student training could help mitigate discussed barriers to curricula implementation, such as by fostering early role-modeling and advocacy of greater education on health-related social needs. Finally, more research on design of standardized curricula and incorporation into residencies is needed. This could ensure that all future emergency physicians have adequate training in working with SWs to optimally address patients’ health-related social needs.

## LIMITATIONS

Our methods section did not search every available database. There may be published data not stored in a public database or unpublished data. As we searched articles published as of February 2021, there may be relevant data that was published after our search.

## CONCLUSION

Despite the prevalence of social workers working as part of the ED team, there remain limited examples in the literature of effective educational collaboration. None of the identified examples directly compared different educational strategies. Of existing educational models, most employ a short didactic model, which is similar to the way other topics are taught to residents. Very limited information exists on educational opportunities involving medical students and SWs. More research would be helpful to inform future standardized curricula. This review summarizes current practices in the literature and identifies areas for future research.

## Supplementary Information



## Figures and Tables

**Figure:**
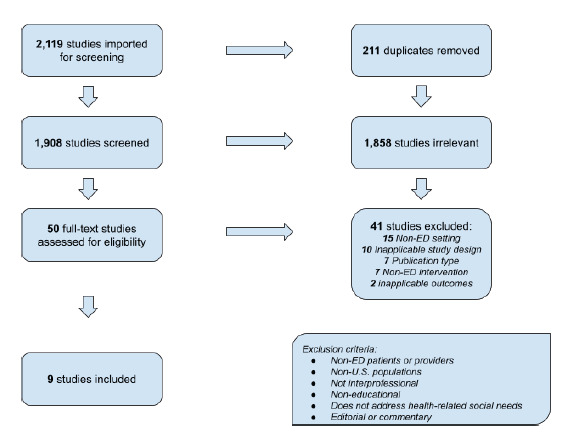
PRISMA Flow Chart *ED*, emergency department; *Non-U.S*, non-United States.

**Table t1-wjem-24-201:** Search strategy.

Curriculum	Students	Social Work	Emergency
“Curriculum”[Mesh] OR “Education, Professional”[Mesh] OR Curricul^*^ OR class OR classes OR course^*^ OR Educat^*^ OR instruct^*^ OR mentor OR school^*^ OR shadow OR skill^*^ OR teach^*^ OR train^*^	“Internship and Residency”[Mesh] OR “Students, Medical”[Mesh] OR student^*^ OR clerkship OR intern^*^ OR resident^*^ OR “house staff”	“Social Work”[Mesh] OR “Social Workers”[Mesh] OR “Community Health Workers”[Mesh] OR “Case Managers”[Mesh] OR “Interdisciplinary Studies”[Mesh] OR social work^*^ OR case manage OR care manage^*^ OR navigator	“Emergency Medicine”[Mesh] OR “Emergency”
