# Supplementary material for: Scoping Review: Medical Education Interventions Optimizing Social Workers in the Emergency Department
Source: West J Emerg Med. 2022 Dec 30;24(2):201–5. doi: 10.5811/westjem.2022.10.55693 (PMC10047740; doi:10.5811/westjem.2022.10.55693)
Supplement: Supplementary file 1 [file wjem-24-201-s001.docx]

**Appendix.**

| **ARTICLE** | **LEAD AUTHOR (YEAR)** | **SETTING AND PARTICIPANTS** | **EDUCATIONAL STRATEGIES** | **DATA COLLECTION METHODS** | **OUTCOMES** | **BARRIERS TO IMPLEMENTATION** |
| --- | --- | --- | --- | --- | --- | --- |
| [Training Emergency Medicine Nurses and Physicians in Youth Violence Prevention](https://www-sciencedirect-com.proxy.hsl.ucdenver.edu/science/article/pii/S0749379705003168) | Cunningham (2005) | 1 Academic ED  EM attendings, residents, RNs | •1 hour case-based, didactic session emphasizing SW  •Distribution of pocket-guide | Pre-intervention self-report survey | •Participants value using the ED to promote violence prevention | Unreported |
| [An evidence-based alcohol screening, brief intervention and referral to treatment (SBIRT) curriculum for emergency department (ED) providers improves skills and utilization](https://www-tandfonline-com.proxy.hsl.ucdenver.edu/doi/abs/10.1300/J465v28n04_01) | Bernstein (2007) | 14 Academic EDs  EM attendings, residents, SWs, RNs, NPs, PAs, EMTs | •2 hour workshop with introductory presentation, 5 video cases, scripted scenarios  •Distribution of pocket-guide | Pre-intervention and post-intervention (3 and 12 month time periods) self-report surveys | •Increased confidence in using SBIRT  •Program well-received by participants | •Time constraints  •Lack of referral resources  •Pressure to preserve patient throughput  •Lack of faculty buy-in |
| [Combating slavery in the 21st century: The role of emergency medicine](https://muse-jhu-edu.proxy.hsl.ucdenver.edu/article/481724) | Chisolm-Straker (2012) | 4 Academic EDs  EM physicians, PAs, RNs, SWs, medical students | •20 minute didactic session | Pre- and post-intervention self-report surveys | •Increased confidence in detecting and responding to human trafficking  •Program well-received by participants | Unreported |
| [Social worker assessment of bad news delivery by emergency medicine residents: a novel direct-observation milestone assessment](https://link.springer.com/article/10.1007%2Fs11739-016-1405-y) | Min (2016) | 1 Academic ED  EM residents, SWs | •Direct observation and feedback by SWs to residents | Post-intervention surveys self-reported by participants and by SW supervisors | •Increased confidence in giving bad news  •Interns valued advice/teaching given by SWs | •Sensitivity of the topic created difficulty in evaluation and assessment of skills |
| [Social workers as workplace-based instructors of alcohol and drug screening, brief intervention, and referral to treatment (SBIRT) for emergency medicine residents](https://www-tandfonline-com.proxy.hsl.ucdenver.edu/doi/full/10.1080/10401334.2016.1164049) | Duong (2016) | 1 Academic ED  1 county trauma center  EM residents, SWs | •1 hour introductory didactic session  •Direct observation and feedback by SWs during regularly scheduled clinical shifts | SWs complete SBIRT checklist and entrustability assessment for residents, recording of encounter time, residents complete satisfaction survey | •PGY1 and PGY2 residents valued the training opportunity  •SWs can be effective at teaching SBIRT during regular clinical shifts | •Time constraints  •Distraction from core clinical responsibilities |
| [A Structured Curriculum for Interprofessional Training of Emergency Medicine Interns](https://www.ncbi.nlm.nih.gov/pmc/articles/PMC6948681/) | Rider (2019) | 1 West Coast ED  EM interns, SWs, RNs, Pharmacists, RTs, LTs | •4 hour shadow shifts and/or didactic sessions with other disciplines | Pre- and post-intervention self-report surveys | •Improved understanding of scope of practice and logistics for different disciplines  •Program well-received by participants | Unreported |
| [Promoting Affirmative Transgender Health Care Practice Within Hospitals: An IPE Standardized Patient Simulation for Graduate Health Care Learners](https://www.ncbi.nlm.nih.gov/pmc/articles/PMC7010321/) | McCave (2019) | ED-based simulation  Students in medical, RN, OT, PT, PA, SW, and health care administration school | •2.5 hour workshop with didactic, simulation, and debriefing sessions | Post-intervention self-report survey | •Improved confidence in interdisciplinary communication  •Program well-received by participants | •Resources required (commitment from trained faculty, simulation space, availability of multiple disciplines) |
| [Interprofessional education of emergency department team on falls in older adults](https://agsjournals-onlinelibrary-wiley-com.proxy.hsl.ucdenver.edu/doi/full/10.1111/jgs.16358) | DeDonato (2020) | 1 Academic ED  EM attendings, residents, nursings, and technicians geriatricians, SWs, PTs | •3 hour didactic session | Pre- and post-intervention self-report surveys | •Improved confidence in ability to care for older adults in the ED | •Complex work schedules of participants  •Time constraints |
| [Point-of-care naloxone distribution in the emergency department: A pilot study](https://academic-oup-com.proxy.hsl.ucdenver.edu/ajhp/article/78/4/360/6130653) | Moore (2021) | 1 Academic ED  EM attendings, residents, RNs, pharmacists | •Development of protocol for naloxone distribution in the ED; training of participants to perform protocol | Tracking of ED naloxone distribution | •Successful distribution of naloxone take-home kits in the ED | •Time constraints  •Distraction from core clinical responsibilities  •Sensitivity of topic |
